# An Evaluation of Healthcare Information on the Internet: The Case of Colorectal Cancer Prevention

**DOI:** 10.3390/ijerph110101058

**Published:** 2014-01-15

**Authors:** Chia-Ching Chen, Tetsuji Yamada, John Smith

**Affiliations:** 1 New York Medical College, 95 Grasslands Road, Valhalla, NY 10595, USA; 2 Rutgers University, the State University of New Jersey, 311 North Fifth Street, Camden, NJ 08102, USA; E-Mails: tyamada@crab.rutgers.edu (T.Y.); smithj@camden.rutgers.edu (J.S.)

**Keywords:** colorectal cancer screening, health information, Internet

## Abstract

Health information, provided through the Internet, has recently received attention from consumers and healthcare providers as an efficient method of motivating people to get screened for colorectal cancer (CRC). In this study, the primary purpose was to investigate the extent to which consumers were better educated about CRC screening information because of the information available on the Internet. Another purpose was to identify how better-informed consumers, with reliable and trustworthy health information, were enabled to make sound decisions regarding CRC screening. The data used in this study was taken from the 2003 Health Information National Trends Survey. People aged 55 and older were classified based on their compliance with recommended CRC screening. The study applied the PRECEDE-PROCEED model to evaluate the effects of health information taken from the Internet regarding CRC screening. The credibility and reliance of cancer related information on the Internet was significantly associated with patient compliance to be screened for CRC. Experience and knowledge of Internet use had a significant impact on the utilization of CRC screening. This analysis suggests that the design and publishing websites concerning CRC should emphasize credibility and reliance. Websites providing information about CRC must also contain the most current information so that people are able to make educated decisions about CRC screening.

## 1. Introduction

The rapid growth in the population of senior citizens with chronic illnesses has generated a marked increase in health care service utilization [1,2,3,4]. Health and medical related information found on the Internet has recently been identified by consumers and health care providers as a powerful means of communication which can improve the health outcomes in all segments of the population [5,6,7,8]. Insurance companies, health professionals, and the pharmaceutical industry encourage the use of various health intervention methods to improve the quality of health, the availability of health information on the Internet [9,10,11]. In the ageing population in particular, the Internet provides a significant means for dispensing important health information [12,13,14,15].

In the United States, more than 70% of patients reported that the health information they found on the Internet influenced their treatment decisions [5,16]. The Internet has emerged as a popular source of health related information and is one of the top two sources (*i.e.*, websites and healthcare providers) [17]. Furthermore, people prefer utilizing the Internet before other methods when seeking information about cancer, and Internet users tend to be younger and better educated adults [18,19]. Thus, the Internet has become a powerful tool for modern day information seeking patients to reduce their health risks and change their health behaviors [15,20,21].

Unfortunately, despite these advantages, unsolicited posting of information on the Internet can cause as much harm as good. Distinguishing reliable information from misleading and inaccurate information is often very difficult for consumers, most of whom have no formal medical education or background [13,19,22,23]. The barriers to reliable information are that anecdotal web pages often have simple and uniformly designed pages that are more convenient for elderly Internet users to navigate [8,24,25]. A decrease in psychological barriers to Internet use is also essential in targeting the growing proportion of older adults in need of health care and health information. Chen, Basch, and Yamada [26] support that greater perceived risk and lower psychological barriers are associated with colonoscopy test use.

The popularity of utilizing the Internet as a source of health information has led to improvements in cancer prevention efforts, particularly among older adults. The Internet can undoubtedly serve as an effective tool in cancer treatment and prevention by pooling information from health professionals and health authorities, as well as providing a useful means through which patients can prompt questions. However, the quality and quantity of health information on the Internet requires further investigation. To date, several studies have attempted to examine the readability of cancer information on websites concerning breast cancer, prostate cancer, colorectal cancer, gynecological cancers, and brain cancer [25,27,28,29]. Barriers to Internet usage [24,30], the purposes of seeking information on the Internet [31,32], and the quantity and accessibility aspects of consumer-oriented health care information [14,33] have been examined, but less so with the focus on cancer-related information. In particular, little is known about how health information on the Internet influences the decisions of people eligible to be screened for colorectal cancer. Promoting the public’s awareness of cancer prevention knowledge and behaviors through the Internet is essential for consumers to improve their quality of life. In this study, the 1st objective was to evaluate how qualitative and quantitative health information on the Internet affects the number of people getting screened for colorectal cancer. The 2nd objective was to evaluate if better-informed individuals, with reliable and trustworthy health information, were more likely to decide to be tested for colorectal cancer. The evidence suggested that health information found on the Internet enables individuals to make better informed decisions regarding cancer screening.

### 1.1. Brief Background of Colorectal Cancer Screening

Colorectal cancer (CRC) is a term used to describe cancer which develops in the tissues of the rectum or the colon. Cancer cells invade nearby tissue and spread through the bloodstream and lymphatic system to other parts of the body [34]. On average, people have a five to six percent chance of developing CRC in their lifetime [35,36,37]. Men and women are equally likely affected by colorectal cancer, but individuals above the age of fifty are at the highest risk [38,39].

CRC screening tests for changes in tissue, cells, or fluids which may indicate the possibility of colorectal cancer, even in the absence of symptoms. CRC prevention has a number of distinct screening modalities, including the fecal occult blood test (FOBT), endoscopic tests, including flexible sigmoidoscopy (FSIG) and colonoscopy (COL) tests, and the double-contrast barium enemas test [39]. The American Cancer Society and the American Gastroenterological Association recommend screening for CRC starting at the age of fifty, using one or more of the following options: annual FOBT, FSIG every five years, and/or COL every ten years [38,40,41,42,43]. Despite the recommendations from these expert groups, the adoption of these guidelines is progressing slowly and testing compliance remains low. Health communication is the art and technique of informing, influencing, and motivating individuals, institutions, and other public audiences about important health issues. The potential for using health communication as a means to increase CRC screening levels among older adults has rightly received greater attention. The growing significance of the association between Internet health communications and patient behavior has resulted in an emerging role for Internet-based health education in health care settings.

## 2. Method

### 2.1. Data Source

Data for this study was drawn from the 2003 Health Information National Trends Survey (HINTS), a nationally representative telephone survey of U.S. adults (aged 18 or older) by the National Cancer Institute from October 2002 to April 2003. The 2003 HINTS was a valuable dataset for understanding the American public’s use of on-line cancer related information and particularly included colorectal cancer knowledge and screening behaviors. Using a random digit dialing (RDD) approach, HINTS completed telephone interviews with 6,369 respondents, with a final response rate of 62.8%. Blacks and Hispanics were oversampled in order to improve the precision of the subgroup analysis. For the current study, we only focused on those 55 or older given they were at the highest risk of developing colorectal cancer and were therefore recommended to undergo regular prevention screenings. After omitting 332 observations with missing values, which account for roughly 15% of total observations, we had a final sample of 1,818 male and female adults with complete information for all variables used in this study.

The guiding conceptual framework for HINTS proposed that consumer-oriented health communication was comprised of two general stages: the awareness stage and the information-seeking stage [44]. During the awareness stage, persuasive media factors included formal (e.g., physicians and other healthcare professionals), informal (e.g., friends and family), and mass communication health information sources (e.g., Internet, TV, magazines, *etc.*). The extent to which the different sources of health communication could influence health awareness depended on various factors, such as socio-demographic characteristics (e.g., age, race, ethnicity, education, income, *etc.*), knowledge and attitude of cancer related risks, and preventive behaviors (e.g., channel attributes, personal ability).

During the information-seeking stage, consumers acted to pull information from in-person and mediated sources. HINTS contained questionnaires that reflected attributes of information-seeking, focusing on an individual’s affordable channel (e.g., availability and accessibility) as well as factors influencing choices of searching for information concerning cancer (e.g., interactivity, credibility, and cost). In particular, HINTS gave attention to Internet users with a series of focused questions to document on-line, health-related activities. In summary, the two-stage process, as outlined in HINTS, influenced the attitudes, beliefs, and knowledge that, in turn, shaped individuals’ health behaviors.

We hypothesized that individuals’ health behavior, compliance with colorectal cancer screening (Table 1), depended on a number of factors, including health information seeking and access, credibility and reliance of the cancer information, interpersonal trust, knowledge about CRC and CRC screening, and perceived risk. Subsequently, we examined the dimensionality and reliability for these variables. Interpersonal trust was measured by six items concerning individuals’ frequency of general perceptions about the quality of communication and degree of trust in health care providers. Each item had 4 response categories (*i.e.*, never, sometimes, usually, and always). Knowledge about CRC and CRC screening was evaluated by asking about knowledge of CRC prevalence, prevention, and screening strategies, including the age to begin FOBT, frequency of FOBT, age to begin SIG/COL, frequency of SIG/COL, regular check, and family history of CRC. Perceived risk of CRC was assessed with four items concerning the perception of cancer risk such as how likely they thought they would develop colon cancer in the future, how likely to get colon cancer as compared with others, how often they worried about colon cancer, and whether any family members had cancer. Each of those three items had three response categories ranging from low, moderate to high, except the last one which respondents answered yes or no. Table 1 contains the definitions of all the variables used in this study.

We performed further analysis to detect the dimensionality and reliability for the variables selected using item reduction method (*i.e.*, factor analysis) in SPSS. The results showed that the initial eigenvalue for all three independent variables (*i.e.*, interpersonal trust, knowledge about CRC and CRC screening, and perceived risk) was greater than 1, suggesting the appropriateness of using them as one-dimensional constructs [45]. The Cronbach alpha for composite variables were 0.70 for interpersonal trust, 0.37 for knowledge about CRC and CRC screening items, and 0.64 for perceived risk. For a dimensionality issue, the eigenvalue of knowledge about CRC screening was greater than one which supported the validity of the composite variable and mitigates the issue of low Cronbach alpha [46].

**Table 1:** Definition and characteristics of CRC related health information (observations = 1,818).

Variables	Definition	Mean	SD
**Dependent Variable**			
Compliance with colorectal cancer screening	When did you do your most recent colorectal cancer screening? 1 = COL in past 10 years, or sigmoidoscopy in past 5 years (64%), or FOBT in past 2 years; 0 = otherwise (34%)	0.65	0.48
**Independent Variables**			
**Cancer Information Seeking and Access (Quantity Oriented Information)**			
Cancer related health information on Internet	The most recent time you looked for information on cancer, where did you look first? 1 = Internet (29%); 0 = others (71%)	0.29	0.45
Did not know where to get wanted cancer related information on Internet	In the past 12 months, you used the Internet to look for health or medical information, but did not know where to find it. 0 = very strongly disagree (60%); 1 = strongly disagree (8%); 2 = somewhat disagree (12%); 3 = somewhat agree (13%); 4 = strongly agree (6%)	0.94	1.32
Took a lot of effort to reach cancer related information on Internet	The most recent time you looked for cancer information on the Internet, it took a lot of effort to get the information you needed. 0 = very strongly disagree (70%); 1 = strongly disagree (4%); 2 = somewhat disagree (9%); 3 = somewhat agree (13%); 4 = strongly agree (4%)	0.73	1.25
General media of cancer related information (magazines, newspaper, radio, and television)	The most recent time you looked for information on cancer by: 1 = magazines, newspaper, radio, or television (18%); 0 = otherwise (82%)	0.18	0.38
Healthcare provider’s cancer information	The most recent time you looked for information on cancer, where did you look first? 1 = healthcare provider (19%); 0 = otherwise (81%)	0.18	0.39
**Credibility and Reliance of the Cancer Information (Quality Oriented Information)**			
Satisfied with the most recent searching experiences of cancer information on Internet	The most recent time you looked for cancer information on the Internet, you were satisfied with the information you found. 0 = very strongly disagree (69%); 1 = strongly disagree (1%); 2 = somewhat disagree (4%); 3 = somewhat agree (14%); 4 = strongly agree (12%)	0.93	1.52
Felt frustrated to search for cancer related information on Internet	The most recent time you looked for cancer information on the Internet, you felt frustrated during your search for the information. 0 = very strongly disagree (69%); 1 = strongly disagree (8%); 2 = somewhat disagree (9%); 3 = somewhat agree (8%); 4 = strongly agree (6%)	0.67	1.20
Confidence of getting information of cancer prevention and early detection on Internet	Regarding receiving information about cancer prevention and early detection on the Internet, how confident are you getting advice or information about cancer if you needed? 0 = very strongly disagree (62%); 1 = not confident at all (1%); 2 = slightly confident (3%); 3 = somewhat confident (11%); 4 = very confident (23%)	1.27	1.75
Easy to understand cancer related health information on Internet	The most recent time you looked for cancer information on the Internet, the information you found was too hard to understand. 0 = very strongly disagree (70%); 1 = strongly disagree (11%); 2 = somewhat disagree (10%); 3 = somewhat agree (6%); 4 = strongly agree (3%)	0.58	1.05
Getting cancer information from the Internet with strong needs relative to other media	Imagine that you had a strong need to get information about cancer. Where would you go first? 1 = Internet (16%); 0 = others (84%) (books, brochures, pamphlets, family, friend/co-worker, healthcare provider, library, magazines, newspaper, radio, telephone information, cancer organization, television, cancer research/treatment facility, others)	0.15	0.36
Internet user with trust in cancer related information from newspapers (interaction term)	Access to the Internet to look for health or medical information for yourself in the past 12 months; and how much would you trust the information about cancer from newspapers? 0 = not at all (53%); 1 = a little (4%); 2 = some (10%); 3 = more (28%); 4 = a lot (5%)	1.23	1.45
Internet user with trust in cancer related information from television (interaction term)	Access to the Internet to look for health or medical information for yourself in the past 12 months; and how much would you trust the information about cancer from television? 0 = not at all (52%); 1 = a little (5%); 2 = some (9%); 3 = more (26%); 4 = a lot (8%)	1.26	1.49
**Enabling Factors**			
Medicare health insurance	Do you have Medicare health insurance? 1 = yes (56%); 0 = no (44%)	0.56	0.49
Availability of healthcare provider	Not including psychiatrists and other mental health professionals, is there a particular doctor, nurse or other health professional that you see most often? 1 = yes (82%); 0 = no (18%)	0.82	0.38
Income	What is your annual household income from all sources? 1 = ≤$35,000 (58%); 2 = >$35,000 and ≤$50,000 (14%); 3 = >$50,000 and ≤$75,000 (13%); 4 = >$75,000 (15%)	1.85	1.13
**Reinforcing Factors**			
Interpersonal Trust	An individual’s trust in healthcare provider from the following six items. 6 = minimum; 24 = maximum	21.25	3.23
Listen carefully to you	During the past 12 months, how often did doctors or other health providers listen to you carefully? 1 = never (2%); 2 = sometimes (11%); 3 = usually (22%); 4 = always (65%)	3.53	0.74
Explain understandable	How often did they explain things in a way you could understand? 1 = never (2%); 2 = sometimes (10%); 3 = usually (23%); 4 = always (65%)	3.53	0.73
Show respect	How often did they show respect for what you had to say? 1 = never (1%); 2 = sometimes (7%); 3 = usually (16%); 4 = always (76%)	3.68	0.64
Spend enough time with you	How often did they spend enough time with you? 1 = never (4%); 2 = sometimes (11%); 3 = usually (24%); 4 = always (61%)	3.45	0.81
Involve you in decision making about your health	How often did they involve you in decisions about your health care? 1 = never (4%); 2 = sometimes (11%); 3 = usually (22%); 4 = always (63%)	3.48	0.81
Trust the cancer information from the doctor	How much would you trust the information about cancer from a doctor or other health professional? 1 = never (3%); 2 = sometimes (6%); 3 = usually (31%); 4 = always (60%)	3.52	0.70
**Predisposing Factors**			
Knowledge about CRC and CRC screening	Knowledge about CRC and CRC screening from the following six items. 0 = minimum point = 1%; 1 = 3%; 2 = 23%; 3 = 32%; 4 = 25%; 5 = 15%; 6 = maximum points = 1%	3.27	1.17
Age to begin FOBT	At what age are people supposed to start doing home stool blood tests? 1 = correct, The answer is “age = 50, or when a health provider says”. (33%); 0 = incorrect (67%)	0.33	0.47
Frequency of FOBT	In general, once people start doing home blood stool tests, about how often should they do them? 1 = correct, The answer is “every 1 ≤ 2years”. (53%); 0 = incorrect (47%)	0.53	0.50
Age to begin SIG/COL	At what age are people supposed to start having sigmoidoscopy or colonoscopy exams? 1 = correct, The answer is “age = 50, or when a health provider says”. (39%); 0 = incorrect (61%)	0.39	0.49
Frequency of SIG/COL	In general, once people start having sigmoidoscopy or colonoscopy exams, about how often should they have them? 1 = correct, The answer is “every 5 ≤ 10 years”. (12%); 0 = incorrect (88%)	0.12	0.32
Regular check	Getting checked regularly for colon cancer increases the chances of finding cancer when it is easier to treat. 1 = correct (89%); 0 = incorrect (11%)	0.89	0.32
Family history with a chance of getting colon cancer	Do you think having a family history of cancer may affect a person’s chances of getting cancer? 1 = correct (92%); 0 = incorrect (8%)	0.92	0.26
Perceived Risk	The sum of absolute risk, relative risk, and cancer worry of the following three variables. 3 = minimum; 9 = maximum	4.41	1.45
Absolute risk	How likely do you think it is that you will develop colon cancer in the future? 1 = low (62%); 2 = moderate (30%); 3 = high (8%)	1.46	0.64
Relative risk	Compared to the average {man/woman} your age, would you say that you are {_} likely to get colon cancer? 1 = less likely (51%); 2 = about as likely (35%); 3 = more likely (14%)	1.63	0.71
Cancer worry	How often do you worry about getting colon cancer? 1 = rarely or never (71%); 2 = sometimes (25%); 3 = all the time and often (4%)	1.33	0.55
Cancer history	Have any of your brothers, sisters, parents, children, or other close family members ever had cancer? 1 = yes (66%); 0 = no (34%)	0.66	0.47
Education			
High school and less	The highest grade or year of school an individual completed. 1 = high school graduate or less (49%); 0 = otherwise	0.48	0.50
Some college	The highest grade or year of school an individual completed. 1 = some college or technical school (29%); 0 = otherwise	0.23	0.42
University and more (omitted variable)	The highest grade or year of school an individual completed. 1 = college 4 years and more (27%); 0 = otherwise	0.26	0.44
**Socio-Demographic Factors**			
Age	What is your age? number of years, minimum = 55, maximum = 95	67.92	9.20
Gender	Are you male or female? 1 = male (37%); 0 = female (63%)	0.36	0.48
Marital status	Are you married, divorced, widowed, separated, never been married, or a member of an unmarried couple? 1 = married (49%); 0 = others (divorced, widowed, separated, never been married, a member of an unmarried couple) (51%)	0.49	0.50
White (omitted variable)	Which one or more of the following would you say is your race? 1 = Non-Hispanic white (78%); 0 = otherwise	0.80	0.40
Black	Which one or more of the following would you say is your race? 1 = Non-Hispanic black or African American (12%); 0 = otherwise	0.10	0.29
Hispanic	Which one or more of the following would you say is your race? 1 = Hispanic (10%); 0 = otherwise	0.07	0.26

Note: SD stands for standard deviation of each variable.

### 2.2. Empirical Framework

**Figure 1:**
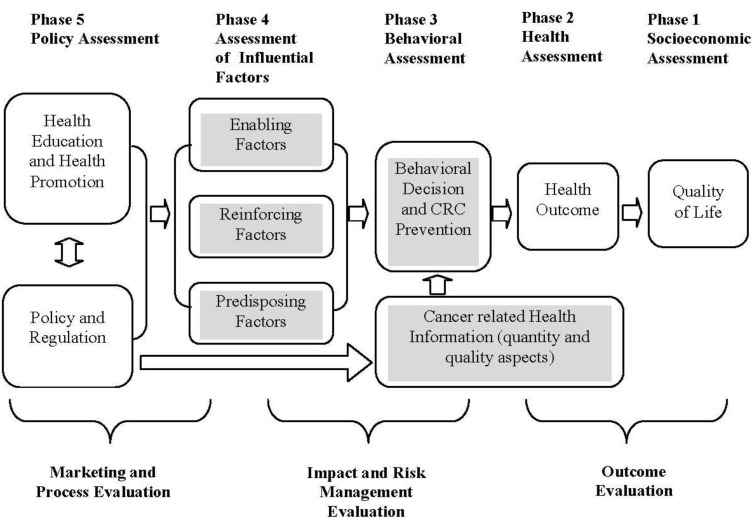
Application of PRECEDE-PROCEED model to internet cancer related health information and colorectal cancer (CRC) prevention.

Note: The PRECEDE-PROCEED framework is adopted from Green and Kreuter (2005) [47].

The study adapted the PRECEDE-PROCEED model (PP model) [47] to understand two aspects of cancer related health information on the Internet: quantity (Internet usage, seeking, and access), and quality (satisfaction, frustration, confidence, reliance, credibility, and trust). Figure 1 shows the adapted PP model in which the Internet health information can have effects on CRC screening behaviors. Generally, the full PP model consists of eight phases: five phases of assessment and three phases of evaluation. Phase 1 and Phase 2 are about socio-demographic and health assessments. Phase 3 includes health behaviors, and in our case, CRC screening. Phase 4 contains three categories of influential factors: the enabling factors, the reinforcing factors and the predisposing factors. The policy assessment (Phase 5) is the assessment of health education and promotion as well as government policy on health information posted on the Internet. The three phases of evaluation in the model generally include marketing and process evaluation, impact and risk management, and outcome product evaluation. For the current study, we were interested in the impact evaluation of the influential factors as shaded in Figure 1. Specifically, we focused on evaluating the effects of Internet usage on colorectal cancer (CRC) screening tests, controlling for other enabling, reinforcing, and predisposing factors.

Our approach to evaluating health information on CRC screening is generally supported by the economics of health behavior, which argues for a demand for health approach to understanding decision-making behavior through the control of the health service sector. According to this theory, health behavior is determined by the production and demand for health, as well as by the institutional framework, *i.e.*, health policy, corresponding regulation, and the related organization [48,49,50,51,52]. This line of research on health behavior and its policy implications has developed along with the PP model which offers useful concepts and analytical tools for analyzing policy influences on behavioral decisions [47,53]. Our empirical study was an extension of the PP model to understand the influence of Internet usage on health behaviors, *i.e.*, CRC screening.

### 2.3. Statistical Model

The study assumed that health was measurable and interpretable as a flow per unit of time. This is because individuals seeking health information possess heterogeneous needs, such as routine and preventative physician care, specialty physician services, acute care, treatment, and health risk. Individual healthcare needs quantitatively and qualitatively are attributed to the physical and environmental domains. These domains are closely related to health outcomes and will impact the quality of life of consumers. This study used Grossman’s concept of separable impact of health education in health capital, and the health status of individuals to analyze physical and environmental domains [49,50,54]. Therefore, the extended PP model incorporated Internet-mediated colorectal cancer screening behaviors with health information seeking behaviors to understand colorectal cancer (CRC) prevention. These decision behaviors are shown in Figure 1.

Enabling factors theoretically include access to health care facilities, availability of resources, referrals to appropriate providers, barriers, and financial sources. Healthy behavior by individuals is generally dependent upon their perception that the information obtained is trustworthy and reliable. The reinforcing factors consist of different types of feedback and rewards for those in the target population after changing their behavior related to colorectal cancer. Predisposing factors characteristically include knowledge, attitude, beliefs, perception, value, ethnicity, and culture. Due to an insufficient level of health literacy and poor health information-seeking abilities, individuals may not know how to evaluate the quality of health information they find on the Internet.

The CRC screening decision, influenced by health information, enabling, reinforcing, and predisposing factors in Phases 3 and 4 (Figure 1) can help improve health outcomes, if individuals are better informed of health information. More accurate health information leads to increased CRC prevention, and the amount of time invested in researching health information on the Internet, influences an individual’s health outcome [48,49]. Accurate and reliable information about cancer allows individuals to make informed decisions about preventive measures as well as treatment decisions by improving their health literacy. Logically, the health information found on the Internet should lend individuals more control over choices of information, knowledge, prevention, screening, treatment, and health risks. Hence, the main hypothesis of the paper was that better-informed individuals, with reliable and trustworthy Internet-based health information, were more likely to get CRC screening.

A functional behavioral model in the shaded areas of Figure 1 could be expressed as the basic estimation equation for the health behavior of an individual:

*CRC_i_* = *α*_0_ + *α*_1_*HI_i_* + *α*_2_*EN_i_* + *α*_3_*RE_i_* + *α*_4_*PR_i_* + *α*_5_*SOD_i_* + *ε_i_* *i* = 1,..., *k* (1)

Equation (1) postulates that an individual’s health behavior of CRC (compliance with colorectal cancer screening) depends on health information HI (cancer information seeking and credibility, and reliance on the cancer information); enabling factors EN (health insurance, availability of healthcare provider, and income); reinforcing factors RE (interpersonal trust), predisposing factors PR (education, knowledge about CRC and CRC screening, perceived risk, and cancer history), and socio-demographic factors of SOD. Socio-demographic characteristics, including age, gender, and marital status, race/ethnicity and other definitions of the variables used in this study are presented in Table 1. The unobserved error, ε, is assumed to satisfy E(ε | HI, EN, RE, PR, SOD) = 0 which means that the error ε has an expected value of zero given any value of HI (health information), EN (enabling factors), RE (reinforcing factors), PR (predisposing factors) and SOD (socio-demographic characteristics). The error, ε, contains all the other factors besides HI, EN, RE, PR and SOD that determine the value of health behavior of CRC (compliance with colorectal cancer screening). An individual’s need for health information is incorporated in the extended PP model to observe their decision-making health behavior and other influential determinants.

## 3. Results

The main dependent variable for this study was whether individuals had a recommended colorectal cancer screening within the recommended time period (*i.e.*, colonoscopy every 10 years, or sigmoidoscopy once every 5 years, or the fecal occult blood test once every 2 years). The empirical results of this study in Table 2 show how influential the quantity and quality oriented cancer-related health information influenced the decision to be screened for CRC.

### 3.1. Cancer Information Seeking and Access

Table 2 presents the results of the probit regression for Internet-based cancer related health information and other factors (definition shown in Table 1). Overall, the results indicated that quantity oriented cancer information seeking from the Internet was among the most important factors for the decision to do CRC screening (estimated coefficient = 3.252). Cancer related health information from the Internet increased individuals’ health knowledge, improved their attitude towards health, and motivated them to further obtain other health-risk information and health services. The higher the level of information seeking on the Internet, the greater the probability an individual had CRC screening. Specifically, 82.6% of individuals who used cancer related health information on the Internet took the colorectal cancer screening (3.252(coefficient) × 0.29 (mean of cancer related health information on Internet) = 0.943 and “*z* value” in the cumulative standard normal distribution was *z* = 0.94 which was about 82.6%). Compared to those who did not use Internet for cancer related information, the Internet users were 66% more likely as indicated by the marginal coefficient (0.660). Interestingly, other information seeking and access variables were not statistically significant although they were in the expected directions. In other words, the analysis suggests that not knowing where to obtain cancer related information and expending a great deal of effort to reach cancer related information are not significant, but have the predicted signs.

**Table 2:** Cancer related health information on the Internet and colorectal cancer screening: the results of Probit regression (N = 1,818).

Variables	Estimate	*p* value	Marginal
**Dependent Variable**			
Compliance with colorectal cancer screening			
**Independent Variables**			
**Cancer Information Seeking and Access (Quantity Oriented Information)**			
Cancer related health information on Internet	3.252 ^b^	0.040	0.660 ^b^
Did not know where to get wanted cancer related information on Internet	−0.200	0.283	−0.041
Took a lot of effort to reach cancer related information on Internet	−0.490	0.129	−0.099
General media of cancer related information (magazines, newspaper, radio, and television)	−0.523	0.290	−0.106
Healthcare provider’s cancer information	−0.526	0.237	−0.107
**Credibility and Reliance of the Cancer Information (Quality Oriented Information)**			
Satisfied with the most recent searching experiences of cancer information on Internet	0.737 ^b^	0.018	0.150 ^b^
Felt frustrated to search for cancer related information on Internet	−0.518 ^c^	0.082	−0.105 ^c^
Confidence of getting information of cancer prevention and early detection on Internet	0.129	0.189	0.026
Easy to understand cancer related health information on Internet	0.470 ^c^	0.092	0.095 ^c^
Get cancer information from the Internet with strong needs relative to other media	−0.798 ^b^	0.016	−0.162 ^b^
Internet user with trust in cancer related information from newspapers	0.695 ^a^	0.004	0.141 ^a^
Internet user with trust in cancer related information from television	−0.431 ^b^	0.036	−0.088 ^b^
**Enable Factors**			
Medicare health insurance	−0.618	0.238	−0.126
Availability of healthcare provider	1.945 ^a^	0.000	0.395 ^a^
Income	0.227 ^c^	0.082	0.046 ^c^
**Reinforcing Factors**			
Interpersonal Trust	−0.049	0.338	−0.010
**Predisposing Factors**			
Knowledge about CRC and CRC Screening	0.465 ^a^	0.000	0.094 ^a^
Perceived Risk	0.107	0.293	0.022
Cancer history	1.266 ^a^	0.001	0.257 ^a^
Education (omitted variable = university and more)			
High school and less	−0.039	0.922	−0.008
Some college	0.349	0.345	0.071
**Socio-Demographic Factors**			
Age	0.042	0.201	0.009
Gender	−0.099	0.722	−0.020
Marital status	−0.319	0.369	−0.065
Black (omitted variable = White)	1.072 ^b^	0.020	0.217 ^b^
Hispanic (omitted variable = White)	−1.526 ^b^	0.025	−0.310 ^b^
Constant	−4.770 ^b^	0.050	−0.968 ^b^
Number of observations = 1,818			
Log likelihood = −1,712.23			
Wald Statistic = 189.30			
Probability > chi-square = 0.0000			
Pseudo R^2^ = 0.0554			

Note: a, b, and c represent statistically significant levels of Probit coefficients as follows: 99% level (a), 95% level (b), and 90% level (c) for a two-tailed test.

### 3.2. Credibility and Reliance of the Cancer Information

Seven items in Table 2, related to the credibility and reliance of cancer information found on the Internet, are included to measure quality oriented information (Definition shown in Table 1). People who were “unsatisfied with their most recent research experiences of cancer information on Internet” tended to have a lower probability of CRC screening. Or specifically, they had a margin of 15% less likely to comply with CRC screening compared to satisfied individuals. For the estimated coefficient −0.737, corresponding probability of 75.49% implied that more than 3 out of 4 individuals who were unsatisfied with the cancer information on the recent Internet search did not take the colorectal cancer screening (−0.737 (coefficient) × 0.93 (mean of unsatisfied with the most recent searching experiences of cancer information on Internet) = 0.6854 and “*z* value” in the cumulative standard normal distribution was *z* = 0.69 which was about 75.49%). The negative coefficient of “felt frustrated to search for cancer related information” in Table 2 shows a margin of 10.5% less likely to comply with CRC screening compared to those who were frustrated with cancer information. Frustrated Internet searchers for cancer related information had a lower probability of complying with CRC screening with a probability of 63.68%. In other words, better designed information source sites and an access to quality oriented cancer related health information induced those people to have CRC screening within the recommended time period. If healthcare professionals were able to recognize these unsatisfied searching experiences, they could present information more effectively on the websites. The fact that the “easy to understand information” coefficient was calculated to have a positive estimate indicated that an individual was more likely to comply with CRC screening with a higher probability of 60.64% and an increase in marginal effect of 9.5% if the sources on the Internet were easy to understand. Accuracy of the contents with clear presentation of cancer related information found on the web sites would also lead to a compliance with CRC screening.

The variable of “Internet usage with trust in cancer information from newspapers” indicated that Internet users who trusted the cancer information they found in newspapers had an increase in CRC screening with a margin of 14.1% in a one scale increment relative to individuals who did not trust the information from newspapers. This implies that not only are Internet sources important in the compliance with CRC screening guidelines, but also the information from newspaper sources is also helpful. The coefficient of “Internet user with trust in cancer related information from television” revealed that individuals with cancer related health information from television tended to reduce their use of Internet for cancer related health information, which in turn translated into a probability (8.8%) of not complying with the CRC screening recommendations.

### 3.3. Enabling, Reinforcing, and Predisposing Factors

Enabling, reinforcing and predisposing factors are the other key policy variables in this analysis. The negative relationship between the government financed Medicare health insurance for retired individuals and CRC screening produced an interesting result: those who were federally insured were less likely to comply with CRC screening than beneficiaries of private health insurance, though the result was not statistically significant (95% of confidence intervals: 0.467~1.027). The statistically significant coefficient of “availability of healthcare providers” revealed that more access to CRC screening services through private health insurance might provide better overall coverage for CRC screening. Another enabling factor, the income variable, was statistically significant, and positively affects the CRC screening among participants ageing 55 or over.

An additional policy variable, defined in Table 1 as the “knowledge about CRC and CRC screening”, was found significant in the predisposing factors. As shown the table, there was a low level of knowledge about CRC and CRC screening nationwide. The positive coefficient with statistically significant regression results implies that formal and informal education, designed to enhance accurate knowledge about CRC and CRC screening, is a crucial feature in increasing CRC screening. In other words, the positive coefficient indicates that colorectal cancer prevention is attributable to health education, which shows that an increase in the knowledge about CRC and CRC screening recommendations are effective in the long run.

## 4. Discussion and Conclusions

This study interweaves health economic theory with the extended PRECEDE-PROCEED model to analyze cancer related health information found on the Internet, and the behavioral decision over CRC screening using the data of the 2003 Health Information National Trends Survey (HINTS). It examined how influential the quantity and quality oriented cancer-related health information affected the amount of people getting screened for CRC so as to develop and publish effective websites for cancer related health information. The study integrated tools of the extended PP model with economic theory of health behavior in order to evaluate compliance with CRC screening.

One of the key policy variables in analyzing this phenomenon has been the quantity of cancer related health information found on the Internet. The findings showed that the amount of Internet usage was associated with a 66% increase in CRC screening compliance, much higher than those who used information from other sources such as newspaper, magazines, *etc.* It should also be noted that there is a possibility that the sources of cancer related health information obtained from television acts as substitute for information found on the Internet, and therefore, reduces information seeking activities on the Internet. Therefore, learning or teaching how to better utilize the Internet for cancer-related health information could be an important policy option for the target senior populations. In this regard, the findings of this study are consistent with Burd, Chiu, and Mcnaught [12] and Tu and Cohen [15].

This study found that dissatisfying and frustrating experiences in searches for Internet-based cancer information could be barriers to getting more people to comply with CRC screening recommendations. The results’ quality oriented information (*i.e.*, credibility and reliance) in this study are consistent with the study by Bremner, Quinn, Quinn, and Veledar [22] and O’Grady [33]. Policy makers need to find ways to more efficiently manage cancer-related information on the Internet; meanwhile, incentives may be necessary for healthcare providers and health insurers to put high quality health information on the Internet. Private insurance could possibly be an effective way to increase the level of compliance with CRC screening within the recommended time period. Through a reduction of dissatisfying experience of seeking cancer-related health information on the Internet, and through improvement of access to information about cancer on the Internet, individuals will be more likely to comply with CRC screening guidelines.

The study also demonstrated that the credibility and reliance of cancer information websites was significantly associated with colorectal cancer screening. In other words, the quality of available health information is an important factor for consumer’s decision making. Credible, reliable, and up-to-date cancer information on the Internet has the potential to increase CRC screening and decrease risk behaviors. In the long run, improvement in prevention efforts and reduction in risk behavior would serve to lower the healthcare expenditures.

Some limitations of the current study need to be considered when interpreting the findings and drawing conclusions. First, the 2003 HINTS data were cross-sectional, indicating the identified relationships needed to be cautiously interpreted as associations rather than causality. Furthermore, the nature of cross-sectional data limited the investigation of behavioral changes across time periods, such as how interpersonal trust between patients and healthcare providers developed, and whether or not these relationships were stable over time. A longitudinal study would provide a more accurate evaluation of the changes over time. Secondly, the estimates of colorectal cancer screening were based on self-reported data and were restricted to the limitations inherent in self-reports. For example, reported increase in colonoscopy test use could be an artifact of a social desirability bias among participants. One possible way to check the reliability and validity of the study is to re-administer the same survey to a random sub-sample one week later to assess the stability of the data. Future efforts must be made to remedy these limitations such as to allow educational and community-based programs to continue to play an integral role in the national health promotion and disease prevention.

Accessibility to healthcare and knowledge of CRC and CRC screening should be major concerns for healthcare delivery. As shown in this study, dispersion of cancer related information can lead to increased knowledge of cancer risk factors for consumers, which in turn would promote CRC and other cancer screening, particularly the elderly members of minority groups, who are at greater risk. Future study should examine the influence of colorectal cancer information of the Internet on the CRC screening behaviors by the minority groups since the actual numbers of African Americans and Hispanic residents participating were relatively low due to the nature of the 2003 HINTS data. It is important to understand the characteristics and to reduce the disparity in the CRC screening among the different ethnicities. Searching for information about CRC can be potentially embarrassing. However, this can be mitigated by searches on the Internet, which can be relatively anonymous.
